# Subperiosteal Schwannoma of the Mid-Tibia: A Cause of Long-Lasting Unexplained Pain

**DOI:** 10.7759/cureus.10269

**Published:** 2020-09-06

**Authors:** Mohamad K Moussa, Christine EL-Yahchouchi, Jean Claude Lahoud, Charbel D Moussallem

**Affiliations:** 1 Orthopedic Surgery, Lebanese University, Beirut, LBN; 2 Anesthesiology, American University of Beirut Medical Center, Beirut, LBN; 3 Orthopedics and Traumatology, Notre Dame Des Secours Hospital, Beirut, LBN; 4 Orthopedic and Spine Surgery, Lebanese University, Beirut, LBN

**Keywords:** peripheral schwannoma, bone tumor, primary neoplastic bone tumor, periosteal schwannoma

## Abstract

Schwannomas are benign tumors affecting the nerve sheath. Their presence in the subperiosteal region is extremely rare. We report a case of a 66-year-old male patient with a 10-year history of unexplained pain of the anterior leg that turned out to be caused by a subperiosteal schwannoma of the mid-tibia. We believe this case report will increase surgeons’ index of suspicion about this condition when dealing with cases of unexplained bony pain, consequently allowing for early diagnosis and better outcomes.

## Introduction

Schwannomas, also known as neurilemomas, are benign tumors that arise from the sheath of peripheral nerves [1,2]. It is a rare condition and usually occurs in soft tissues [3]. In extremely rare cases, schwannomas can present on the bone surface as subperiosteal neoplasm, and this rare condition has been described and reported in only five documented cases so far [4-8]. Herein, we present the sixth case report documenting this rare condition. It involves a 66-year-old man with subperiosteal schwannoma at the anterior aspect of the left mid-tibia that resulted in a decade of unexplained leg pain followed by a two-year period of painful mass.

## Case presentation

A 66-year-old white Mediterranean patient consulted our team due to a painful swelling on the anterior aspect of his distal left tibia. His pain had started about 10 years ago, and two years ago, he had noted some swelling and tenderness at the same location of the pain.

**Figure 1:**
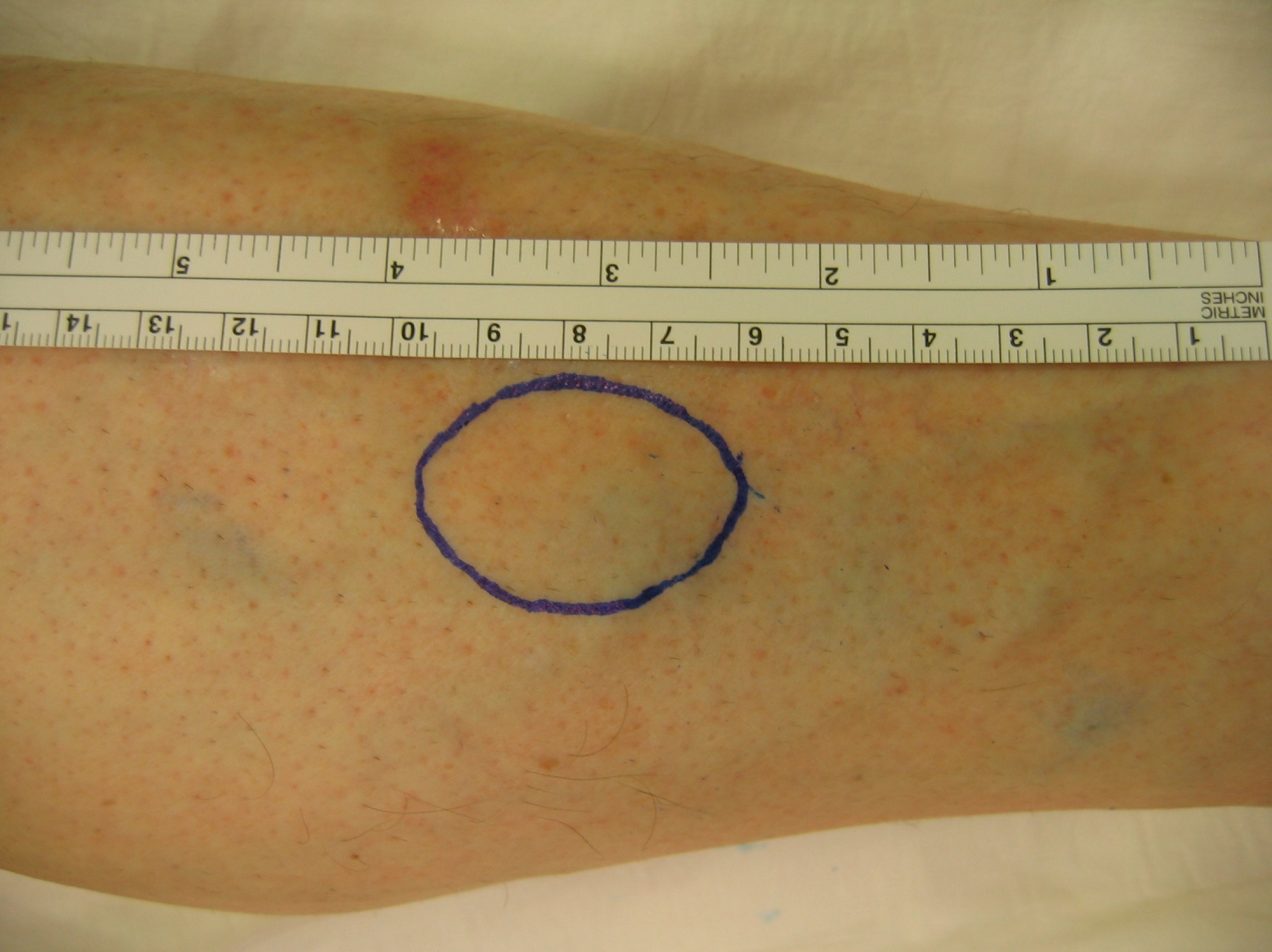
Photo of the leg showing round, well-circumscribed mass on the medial anterior aspect of the mid-tibia (blue circle)

During the physical examination, a 4-cm, well-demarcated mass on the medial anterior aspect of his left tibia (Figure 1) was noted. It was round in shape, fixed to the bone, and covered by normal-appearing skin. The mass was very tender with a lancinating pain on finger tap. The overall physical examination was unremarkable; no other similar lesions were found and no café-au lait spots or cutaneous neurofibroma were noted.

**Figure 2:**
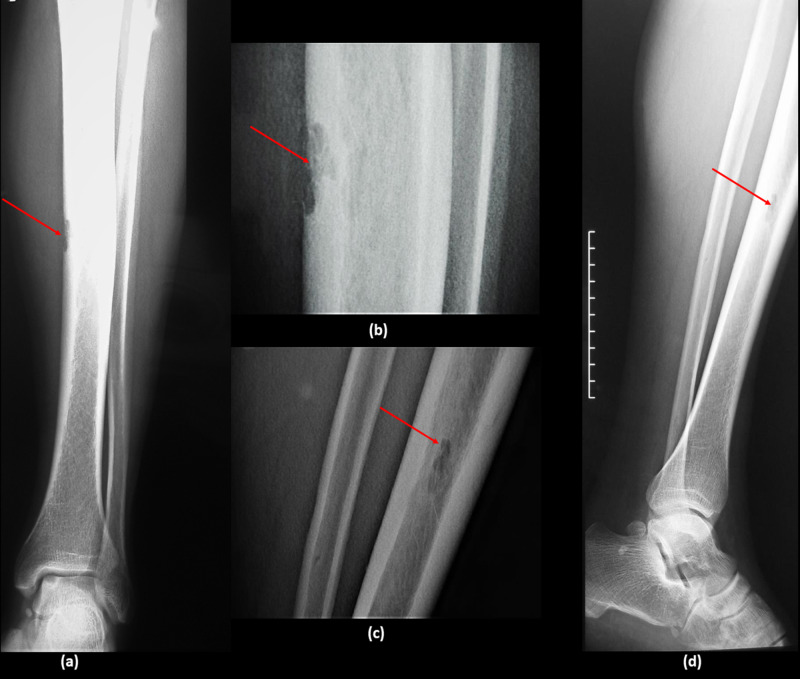
Radiograph of the leg Anteroposterior (a, b) and lateral (c, d) radiographs of the left leg showing evidence of a lytic, geographic, subperiosteal lesion on the medial anterior aspect of the mid-tibia (red arrows)

All laboratory findings including blood count, hematocrit, serum electrolytes, liver function tests, and coagulation tests were normal. Anteroposterior and lateral radiographs of the leg were done and showed evidence of a lytic, geographic, subperiosteal lesion on the medial side of the mid-tibia (Figure 2).

**Figure 3:**
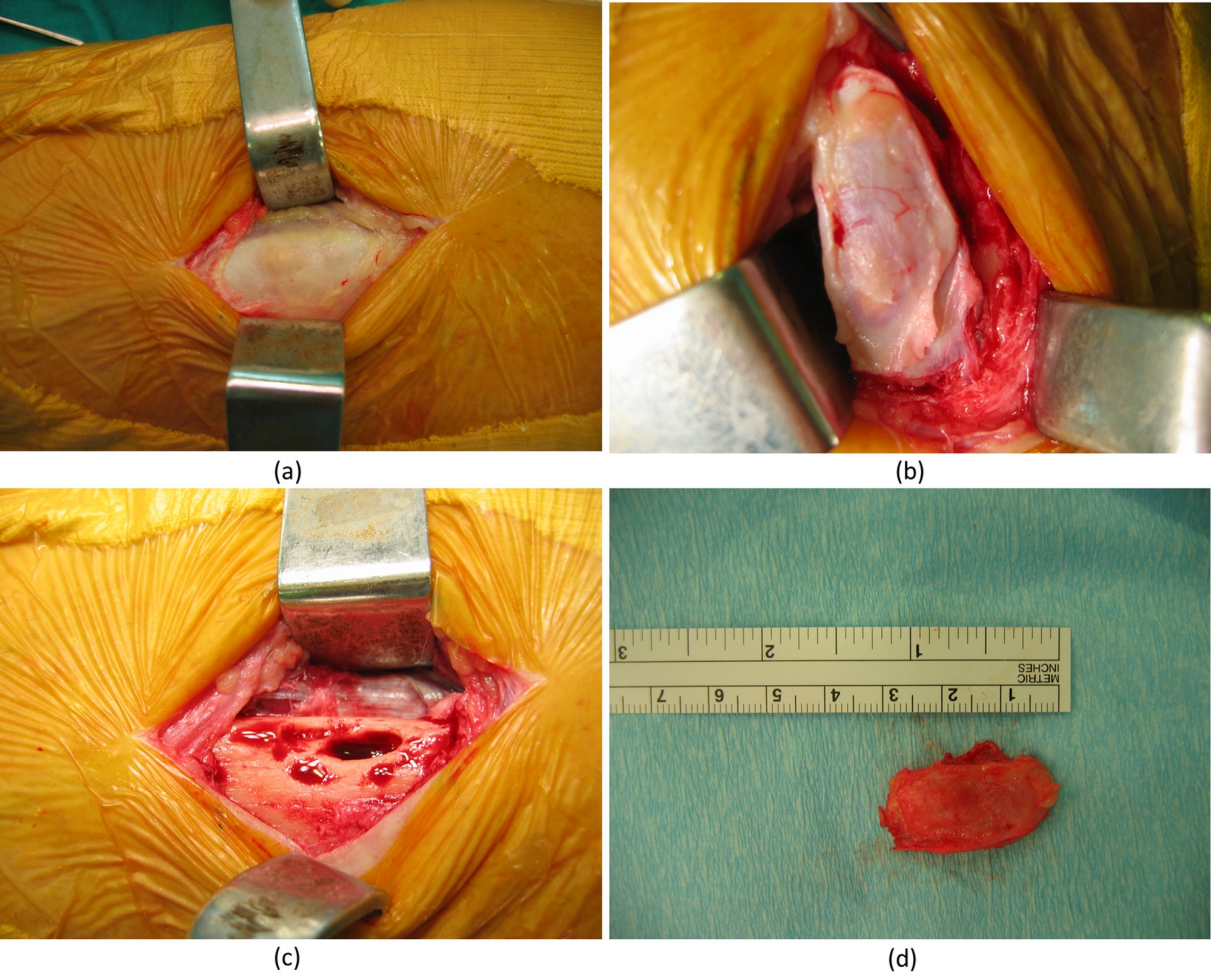
Important stages of surgical intervention (a) incision and subcutaneous dissection till reaching the mass; (b) dissection of the mass from the surrounding tissues; (c) en bloc excision of the mass; (d) the excised 3-cm mass

An MRI was ordered and it showed a juxtacortical lesion that is isointense on T1- and hyperintense on T2-weighted images. The decision was made for a complete surgical excisional biopsy. For this, the patient was taken to the operation room, and a longitudinal incision was done along the lesion. The subcutaneous tissue was dissected and it had a normal appearance. The lesion appeared to be embedded in the cortex (subperiosteal) of the anterior aspect of the tibia. It was well-circumscribed and encapsulated without the involvement of surrounding tissues. It was excised entirely as one piece and was sent to the pathology laboratory for histological studies and culture. The wound was then closed. Figure 3 shows the important stages of surgical intervention.

The postoperative period was uneventful, and the patient was discharged home a day after surgery.

The laboratory results showed a sterile culture. The hematoxylin and eosin-stained sections revealed a subperiosteal benign schwannoma that was completely excised. The pathology report mentioned the presence of a well-encapsulated lesion formed of spindle cells in addition to thin wavy spindle nuclei and nuclear palisading (Verocay bodies). There was no mitosis or necrosis area. Immunohistochemistry staining showed diffuse expression of S100 proteins.

The patient's pain disappeared and he is still free of symptoms at the operation site 13 years after the surgery.

## Discussion

Tumors of the peripheral nerve sheath are relatively uncommon neoplasms in the general population [9]. They can range from benign tumors including neurofibroma, perineurioma, and schwannomas, to malignant tumors, with multiple hybrid benign phenotypes mentioned in the literature [9].

Of these subgroups, schwannoma, which is more properly known as neurilemomas, is the most common tumor of peripheral nerves, usually affecting people aged between 20-50 years of age [10]. They are benign by definition, but some cases of malignant transformation have been reported [11]. Schwannomas may be found in different locations; most of them are within soft tissues. In a review of 234 cases, Knight et al. reported that 170 tumors (73%) were found in the upper limb, 64 (73%) in the lower limb, and only six (2.6%) were located within muscles or bone [3].

The intraosseous location of schwannomas is occasional and may affect any bone of the body, especially the mandible, which is classified as the most commonly affected location where schwannomas arise from the mandibular nerve [12,13]. Fawcett et al. studied 3,987 primary bone tumors at the Mayo Clinic and found only six intraosseous neurilemomas without any case of subperiosteal schwannomas [14].

The first case of subperiosteal schwannoma was reported by Verma et al. in 2002 when they described two adjacent diaphyseal femoral subperiosteal schwannomas in a 38-year-old man, who had been suffering from thigh weakness and pain [4] for four years. The second case was reported by Singh et al. in 2005, when they reported a subperiosteal schwannoma arising from the surface of the diaphysis of the ulna in a 28-year-old female patient that showed up as painful swelling [5]. Afterward, only three more case reports of this disease have been published: in 2014, 2016, and 2020 [6-8]. Table 1 summarizes the different articles reporting this extremely rare location of schwannomas.

**Table 1:** Summary of cases of subperiosteal schwannomas reported in the literature

Authors	Age of the patient, years	Sex	Location	Year of publication	Chief complaint	Time to presentation
Verma et al. (2002) [4]	38	Male	Femur	2002	Pain with no swelling	4 years
Singh et al. (2005) [5]	28	Female	Ulna	2005	Painful swelling	Long duration (nonspecified)
Patro et al. (2014) [6]	35	Male	Femur	2014	Painful swelling	2 years
Lakhotia et al. (2016) [7]	34	Male	Pelvis	2016	Pain with no swelling	3 months
Patro et al. (2020) [8]	35	Male	Proximal tibia	2008	Pain with no swelling	2 years
	30	Male	Femur	2015	Painful swelling	18 months
	45	Male	Proximal tibia	2016	Painful swelling	14 months
Present case (2020)	66	Male	Mid-tibia	2007	Pain alone then painful swelling	10 years

Pain was a constant and chief complaint in all reported cases, and most patients had delayed diagnosis. Interestingly, all cases reported in the literature involved patients around 30 years of age. Our case is the first case to be presented at 66 years of age, which is a very unusual age of presentation, especially given that schwannomas in general show up between 20-50 years of age [10]. Furthermore, this is the third case of periosteal schwannoma that involves the tibia.

Imaging is the most important diagnostic tool for the evaluation of patients with suspected schwannomas [15]; the radiographs can help in localizing the lesion with subperiosteal or intraosseous localization. The radiographic appearances of periosteal schwannomas mentioned in the available case reports are variable; the most constant feature is the presence of a lytic lesion, which was mentioned in nearly all reported cases including our case [4,5,7,8]. Patro et al. have reported a unique case of subperiosteal schwannoma with normal-appearing radiographs despite the long and extended course of their patient's disease (two years) [6]. In addition, MRI helps with the evaluation of the mass and the determination of its relation to the nerve sheath and the surrounding tissues [10]. 

Differential diagnoses of schwannomas include fibroma, neurofibroma, neurosarcoma, ganglion cyst, and lipoma. The gold standard method to make an accurate diagnosis is a surgical biopsy that should be evaluated by a pathologist with expertise in peripheral nerve tumors [16]. Genetic testing for neurofibromatosis types 1 and 2 could be helpful to complete the workup but is not necessary as the concomitant occurrence of schwannomas and neurofibromatosis is rarely reported [17].

All cases of subperiosteal schwannomas, including our case, were treated with complete surgical excision and had favorable outcomes. However, data about the recurrence rate is lacking.

## Conclusions

Although extremely rare, schwannomas or neurilemomas should be considered as a possible differential diagnosis for surface lesions of the bone. As for the clinical aspect and presentation of this disease, the diagnosis should also be considered when a patient has had unexplained localized vague pain and swelling for a prolonged period of time. Surgical excision remains the best-proposed method of treatment by far.
